# Broadband metasurface holograms: toward complete phase and amplitude engineering

**DOI:** 10.1038/srep32867

**Published:** 2016-09-12

**Authors:** Qiu Wang, Xueqian Zhang, Yuehong Xu, Jianqiang Gu, Yanfeng Li, Zhen Tian, Ranjan Singh, Shuang Zhang, Jiaguang Han, Weili Zhang

**Affiliations:** 1 Center for Terahertz waves and College of Precision Instrument and Optoelectronics Engineering, Tianjin University and the Key Laboratory of Optoelectronics Information and Technology (Ministry of Education), Tianjin 300072, China; 2 Center for Disruptive Photonic Technologies, Division of Physics and Applied Physics, School of Physical and Mathematical Sciences, Nanyang Technological University, 21 Nanyang Link 637371, Singapore; 3 School of Physics and Astronomy, University of Birmingham, Birmingham B15 2TT, UK; 4 School of Electrical and Computer Engineering, Oklahoma State University, Stillwater, Oklahoma 74078, USA

## Abstract

As a revolutionary three-dimensional imaging technique, holography has attracted wide attention for its ability to photographically record a light field. However, traditional phase-only or amplitude-only modulation holograms have limited image quality and resolution to reappear both amplitude and phase information required of the objects. Recent advances in metasurfaces have shown tremendous opportunities for using a planar design of artificial meta-atoms to shape the wave front of light by optimal control of both its phase and amplitude. Inspired by the concept of designer metasurfaces, we demonstrate a novel amplitude-phase modulation hologram with simultaneous five-level amplitude modulation and eight-level phase modulation. Such a design approach seeks to turn the perceived disadvantages of the traditional phase or amplitude holograms, and thus enable enhanced performance in resolution, homogeneity of amplitude distribution, precision, and signal-to-noise ratio. In particular, the unique holographic approach exhibits broadband characteristics. The method introduced here delivers more degrees of freedom, and allows for encoding highly complex information into designer metasurfaces, thus having the potential to drive next-generation technological breakthroughs in holography.

The explosive field of metamaterials and metasurfaces has recently provided extraordinary capabilities to control electromagnetic waves as desired and has shown numerous intriguing phenomena and promising applications, including negative and zero index refraction^1^,^2^,^3^,^4^, invisible cloaking^5^,^6^,^7^, super-resolution imaging^8^,^9^, electromagnetic induced transparency^10^,^11^, and topological insulators^12^,^13^. For bulk metamaterials, however, the fabrication challenges and material losses are usually significant and inevitable, which thus hinders their realistic applications. Recently, ultrathin designer metasurfaces were proposed for exerting unprecedented control over the wavefront of electromagnetic waves. The subwavelength thickness of metasurfaces has significantly lower the optical losses along with reduced complexity of the fabrication process. Therefore, metasurfaces have opened an unprecedented avenue for shaping the wavefront of the transmitted radiation and thus offer fascinating properties that could usher in revolutionary application oriented photonic devices, such as high resolution holograms^14^,^15^,^16^,^17^,^18^,^19^,^20^,^21^, ultrathin flat lenses^22^,^23^,^24^, anomalous reflection and refraction^25^,^26^, and quarter wave plates^27^,^28^. The application space of metasurfaces nearly covers the entire electromagnetic spectrum, including the technologically important terahertz regime^29^.

Holography, a technique enabling three-dimensional (3D) imaging, has continuously aroused tremendous curiosity, since the time it was discovered in 1947^30^. Traditional holography works by recording the interference patterns of the light scattered by the object and a coherent beam, which contain both the amplitude and phase information of the wavefront of the scattered light for reconstructing the 3D image^31^. However, the difficulty in adopting this technique arises since it requires real objects and highly temporal and spatial coherent light sources. Later on, a new approach for generating holograms was proposed, which included using numerical computation to calculate the phase distribution at the hologram interface, and coding this information into specific surface structures or a spatial light modulator (SLM). This approach is widely known as computer-generated holography (CGH)^32^. New schemes where metasurfaces have been used as holograms have aroused a lot of attention in the photonic and plasmonic community^14^,^15^,^16^,^17^,^18^,^19^,^20^,^21^. In most of the recent demonstrations, only the phase information has been engineered while the amplitude is kept constant, resulting in an inevitable limitation in obtaining high-resolution perfect images.

In this article, we propose a novel scheme of a broadband metasurface hologram in which we simultaneously tailor the five-level amplitude and eight-level phase modulation at terahertz frequencies. By adopting the C-shape split-ring resonators (CSRRs) as the basic unit structures, our hologram design possesses a higher degree of robustness and broadband characteristics with high resolution and no conjugate image. The hologram is experimentally characterized using near-field scanning terahertz microscopy (NSTM) for its high resolution. We compare the performance of our design with a similar sample with eight-level phase modulation and a constant amplitude. The advantages of simultaneous amplitude and phase control are demonstrated by numerical calculation, 3D full wave simulation and experimental validation. In comparison to the previous CGH designs, this approach has unique advantages including higher precision, reduced noise, and simplified fabrication process. The proposed metasurface hologram design with simultaneous amplitude and phase modulation broadens the horizon of conceiving new routes to realize perfect holography, which may have immense application potentials in terahertz imaging.

## Results

**Figure 1:**
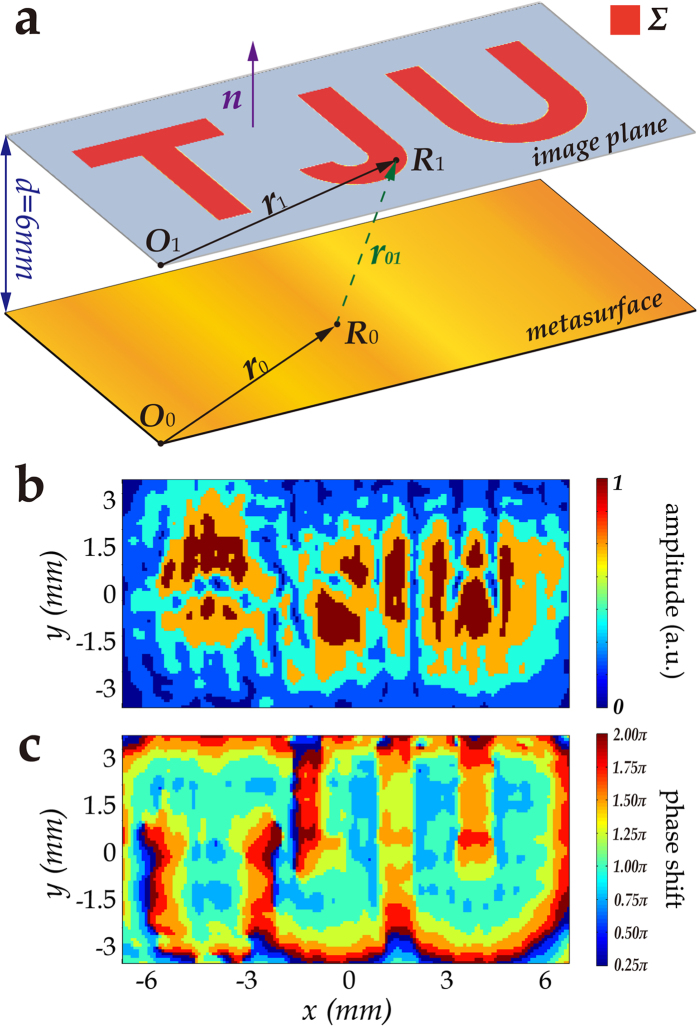
Design of the hologram metasurface. (**a**) Schematic illustrating the calculation of the electric field distribution. The image plane is set at a given position (*d* = 6 mm), where the virtual object (“TJU”) is located. A plane wave incidence is assumed to transmit from the top and pass through the “TJU” aperture. The electric field of the “TJU” region is endowed with the same amplitude and phase so that the required electric field on the metasurface can be calculated by the diffraction theory. (**b**) Calculated five-level amplitude distribution of the electric field on the metasurface after normalization and discretization. (**c**) Calculated eight-level phase distribution of the electric field on the metasurface after discretization.

To design the metasurface hologram, we assume a virtual object on the image plane with *d* = 6 mm away from the metasurface, as illustrated in Fig. 1a. The word “TJU” is taken as the virtual object, which is considered as a flat screen. In the far-field, the Rayleigh-Sommerfeld diffraction theory can be applied for the calculation of the electric field distribution of the metasurface,


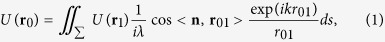


where *U*(***r***_0_) and *U*(***r***_1_) represent the electric fields at point *R*_0_ on the metasurface and point *R*_1_ on the image plane, respectively; Σ is the virtual object region; *λ* is the wavelength in vacuum; ***n*** is a vector perpendicular to the image plane with the orientation shown in Fig. 1a; *r*_01_ is the distance between *R*_0_ and *R*_1_; and cos <**n**, **r**_01_> is the inclination factor. We discretize the virtual object into grids so that the integral transforms into the superposition of many point sources. We set the object with uniform amplitude and phase, so we can let *U*(***r***_1_) = *A*. Thus, the electric field distribution of the metasurface can be expressed in a simpler form as


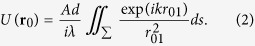


By normalization and discretization, a five-level amplitude distribution and an eight-level phase distribution of the electric field on the metasurface are obtained, as shown in Fig. 1b,c. Linear partition method was applied in the discretization. According to the reversibility of beam propagation, the “TJU” image would reappear on the image plane when the required phase and amplitude distribution of the metasurface is achieved.

**Figure 2:**
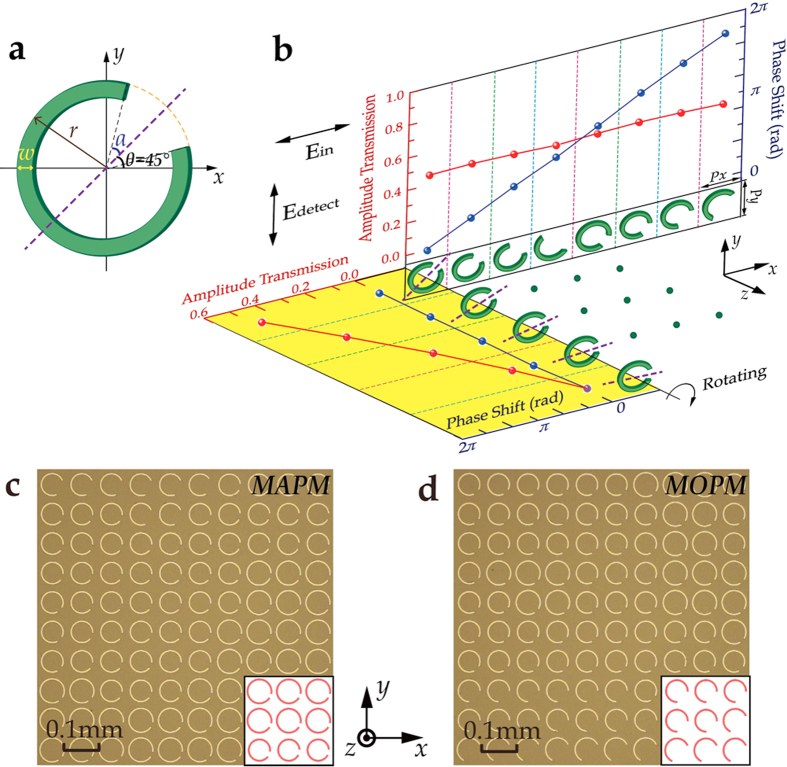
Basic functional units and partial optical images of the metasurfaces. (**a**) Schematic of a CSRR with outer radius *r*, width *w*, opening angle 2*α*, and orientation angle *θ* with respect to the *x*-axis. (**b**) Simulated transmission amplitude modulation and phase shift of the CSRRs at 0.8 THz according to their *r, α* and *θ* parameters. The transmission electric field is *y*-polarized and the incidence is *x*-polarized. The periodicity of the unit cell along *x* and *y* direction is *P*_*x*_ = 80 μm and *P*_*y*_ = 80 μm, respectively. For the upper eight CSRRs in the white graph, *r* and *α* are varying, while *θ* remains 45° or −45°. For the five CSRRs in the yellow graph, however, *r* and *α* remain constant and *θ* is varying, where the amplitude follows a |sin(2*θ*)| dependence with the phase shift remaining invariant. The purple dash lines represent the symmetry axis of the CSRRs. The nine green dots in the middle are the apostrophes representing that similar rotation rules illustrated in the yellow graph also apply to the other seven CSRRs in the white graph. (**c**) Partial optical image of MAPM with both a five-level amplitude modulation and an eight-level phase modulation. (**d**) Partial optical image of MOPM with an eight-level phase modulation and a constant amplitude. The insets on the lower right of **c** and **d** represent the designed sizes of CSRRs.

To realize the metasurface hologram, CSRRs are employed as the basic unit structures to constitute the metasurface with both amplitude and phase information, as shown in Fig. 2a. CSRRs have been widely used in terahertz metasurface design for their strong response to the terahertz radiation and ease in fabrication. For the *x*-polarized incidence, the energy partially converts into a *y*-polarized component in the resonance frequency range. The amplitude and phase of the outgoing *y*-polarized wave can be simultaneously manipulated by varying the geometrical parameters of the CSRRs. Through numerical simulation, eight resonators comprising a phase shift ranging from 0 to 2π and a nearly constant transmission amplitude are chosen to operate at 0.80 THz, as shown in the upper eight CSRRs in Fig. 2b, where *P*_*x*_ = 80 μm and *P*_*y*_ = 80 μm. Furthermore, it has been demonstrated that when the orientation angle *θ* is varied from 0° to 90° while *r* and *α* remain constant, the amplitude of the outgoing *y*-polarized component follows a simple |sin(2*θ*)| dependence, while the phase shift remains invariant^33^, as shown in the lower left in Fig. 2b. This facile way of controlling the amplitude without interfering with the phase profile greatly simplifies the design. Constituted by these unit cells, the metasurface is finally designed to have field distributions as shown in Fig. 1b,c. The metasurface has an overall size of 12.88 mm × 6.48 mm, that is 161 × 81 pixels. The line width of each letter in the word “TJU” is a constant, 0.8 mm.

The metasurface hologram sample is fabricated on a silicon substrate by conventional photolithography. With simultaneous five-level amplitude modulation and eight-level phase modulation, we address this sample as “MAPM” (metasurface with simultaneous amplitude and phase modulation), whose optical image is shown in Fig. 2c. For a comparison, a reference sample that we address as “MOPM” (metasurface with only phase modulation) is designed with the same eight-level phase modulation, whereas the amplitude is kept constant. The maximum constant amplitude is obtained for a fixed orientation of resonators oriented at an angle of 45° or −45°, as shown in Fig. 2d.

**Figure 3:**
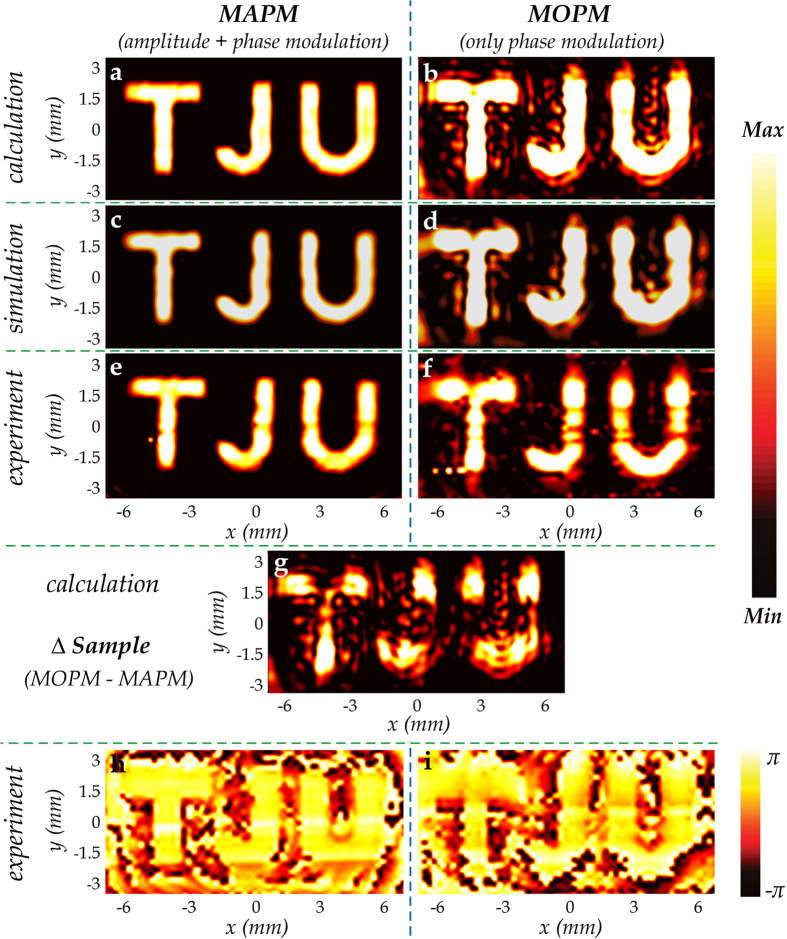
Holographic imaging effects of the MAPM and MOPM samples. (**a,b**) Numerically calculated electric field amplitude distributions on the image plane at 0.8 THz by MATLAB, corresponding to MAPM and MOPM, respectively. (**c,d**) 3D full wave simulated results of amplitude distributions in the *y*-polarization of MAPM and MOPM at 0.8 THz on the image plane, respectively, for the *x*-polarized wave at normal incidence. (**e,f**) Experimental results of the *y*-polarized amplitude distributions on the image plane at 0.8 THz, corresponding to MAPM and MOPM, respectively. The incident wave is *x*-polarized. (**g**) Numerically calculated amplitude distribution of Δ sample on the image plane at 0.8 THz by MATLAB. Δ sample, which can be simply considered as subtracting MAPM from MOPM, is established to explain the differences of the imaging effects between MAPM and MOPM. (**h,i**) Experimental results of the y-polarized electric field phase distributions on the image plane at 0.8 THz, corresponding to MAPM and MOPM, respectively.

The reconstruction of “TJU” on the image plane is first numerically examined by a simple scalar calculation without considering the detailed CSRR geometry,


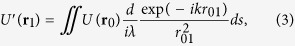


where *U*′(***r***_1_) represents the electric field at point *R*_1_ on the reconstructed image; *U*(***r***_0_) is the electric field at point *R*_0_ on the metasurface after discretization; and the exponential assumes an opposite nature due to the change in the transmission direction of the beam. The calculated results for MAPM and MOPM are shown in Fig. 3a,b, respectively, which reveal tremendous advantages of the MAPM due to simultaneous recovery of both amplitude and phase information. As the image quality relies on the amplitude and phase modulation, metasurfaces with simultaneous control over different level amplitude and phase modulation does not deviate much compared to the phase modulation case in every pixel, which eventually enables a superior quality of the images.

The image reconstruction of the proposed metasurface hologram is further simulated using 3D full wave solver by CST Microwave Studio. The simulation was carried out with the time domain solver and perfectly-matched-layer (PML) boundary conditions are applied in the *x, y* and *z* directions. The spatial grid size is adaptive by the default setting. Normally incident *x*-polarized plane wave is used to excite the structure, and the scattered *y*-polarized radiation is recorded. The simulation results are shown in Fig. 3c,d, corresponding to MAPM and MOPM, respectively. Again, the simulated results highlight the importance of both the amplitude and phase information.

**Figure 4:**
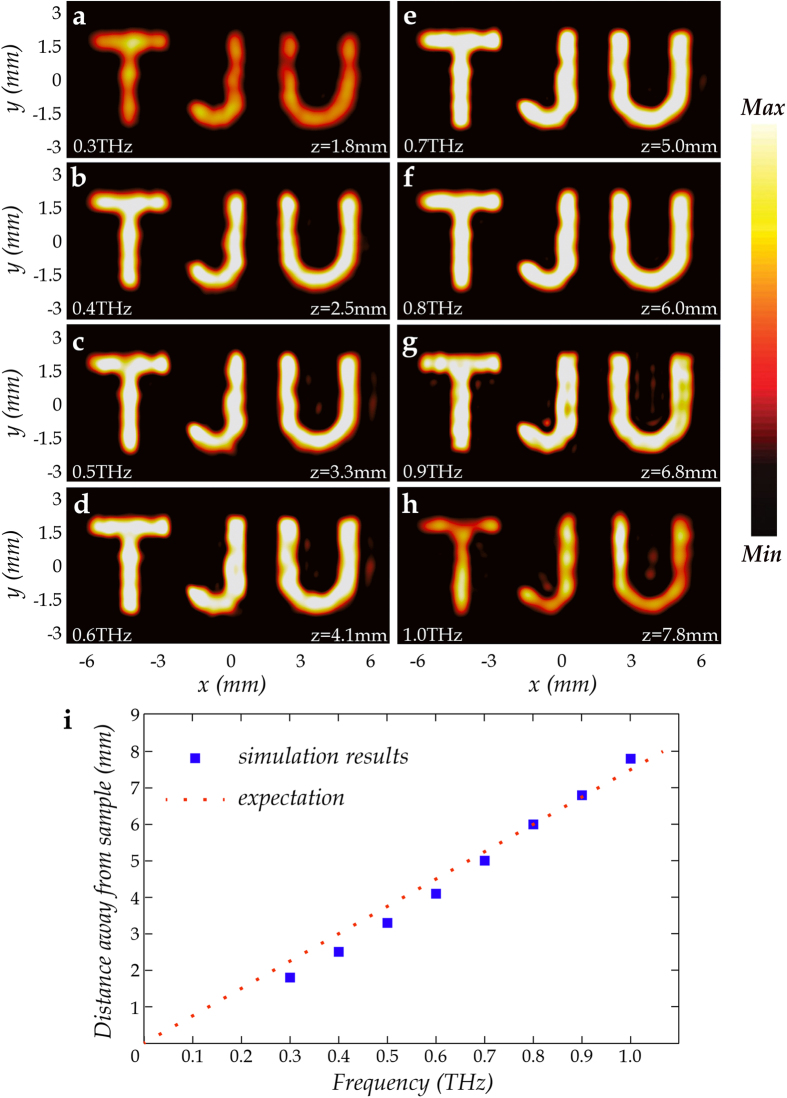
Simulation results for the broadband holographic imaging of MAPM. (**a–h**) Three-dimensional full wave simulation results of electric field amplitude distributions of MAPM at the respective corresponding image planes at different frequencies. At a single frequency, the image plane is determined by the appearance of the best image in the simulation. A broadband imaging performance can be clearly seen from 0.3 to 1.0 THz at the respective corresponding image planes. However, the efficiency slightly decreases when it comes to 0.3 or 1.0 THz. (**i**) Relationship between frequency and *z* (distance away from the sample). The blue square dots represent the simulation results corresponding to (**a–h**) The red dotted line passes through the origin and point (0.8, 6.0), representing the expected proportional relationship between frequency and *z*.

Besides, the 3D full wave simulation results show a broadband functionality of the proposed design method. The simulated electric field amplitude distributions of MAPM at the corresponding image planes at different frequencies are shown in Fig. 4a–h. At a single frequency, the position of the corresponding image plane is determined by the appearance of the best image in the simulation results. An excellent broadband imaging performance can be clearly observed from 0.4 to 0.9 THz. This essentially relies on the broadband property of the CSRRs. They maintain nearly the same amplitude and the same phase shift as the frequency varying in the frequency range mentioned above, which is discussed in detail in our previous work^26^. Slightly out of this range at 0.3 and 1.0 THz, similar effects can also be observed, however, the efficiency slightly decreases. It is worth noting that the distance between the metasurface and the image plane increases with increasing frequency. This can be explained by the different phase accumulation during the wave propagation at different frequencies. For example, we consider point *A (x*_0_, *y*_0_, *z*) as a particular point in the holographic image; point *A*' (*x*_0_, *y*_0_, 0) is the corresponding point on the metasurface; point *M (x, y*, 0) is an arbitrary point on the metasurface. The broadband character is assumed to be ideal. So, for a given point *M*, the phase difference can be easily obtained


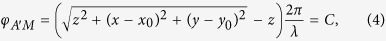


where *φ*_*A*'*M*_ represents the phase difference between point *M* and *A*'; *λ* is the wavelength in vacuum; C is a constant. Let (*x*−*x*_0_)^2^ + (*y*−*y*_0_)^2^ = *a*, where *a* is a non-negative constant, then, equation (4) can be expressed as


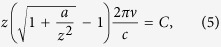


where *ν* is frequency and *c* is the speed of light in vacuum. Under the paraxial approximation and by use of the Taylor formula, the relation can be simplified as


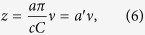


where *a*' is another constant. As the hologram is designed at 0.80 THz with *z* = 6 mm, *a*' is known. Equation (6) indicates that the distance between the metasurface and image plane is approximately proportional to frequency, which agrees well with the simulation results, as shown in Fig. 4i. The slight deviation mainly arises from the fact that the paraxial approximation cannot be met well and CSRR cannot have identical response in a large frequency range.

Experimentally, the two metasurface hologram samples were characterized by use of an NSTM system^24^,^34^. Figure 3e and f show the measured amplitude distribution of the electric field at 0.8 THz on the image plane for MAPM and MOPM, respectively. The experimental results, including the profiles, dimensions, and line width of the image and relative amplitude distributions are in very good agreement with the numerical calculation results by MATLAB and the 3D full wave simulation results by CST for both samples. The deviation in fabrication and experiment leads to the slight difference between the 3D full wave simulation and the experimental results. In terms of all the six figures in Fig. 3a–f, we observe that the results near the image position of MAPM show clearer and sharper image profiles, and much more homogeneous and structured amplitude distribution than those of MOPM, which signifies higher resolution and better imaging quality, respectively. Besides, the littery spots in the results of MOPM reflect that the simultaneous amplitude and phase modulation method provides higher accuracy and lower noise, which are extremely important in evaluating the image quality and efficiency. These results clearly demonstrate the applicability and superiority of the proposed metasurface-based holograms with simultaneous amplitude and phase modulation.

## Discussion

The superiority on the imaging effect of MAPM over MOPM can be clearly seen by comparing the results of MAPM with the blurred image of MOPM, which is formed due to the constant amplitude of MOPM. In the design, the phase distribution of MAPM and MOPM is identical and every pixel of MOPM does not have a lower amplitude than that of the corresponding pixel of MAPM. To explain the differences of the imaging effects between MAPM and MOPM directly, Δ sample, which has the same phase distribution as MAPM and MOPM, and an amplitude distribution that can be considered as a difference between MAPM and MOPM, is established. Numerically calculated amplitude distribution of Δ sample on the image plane at 0.8 THz by MATLAB is shown in Fig. 3g. According to equation (3), the amplitude distribution of MOPM on the image plane should be the sum of that of MAPM and Δ sample. It can be clearly seen that the littery spots and the deviation from the ideal image, especially nearby the boundaries of the words are large, as shown in Fig. 3g, which leads to declined resolution and imaging quality of MOPM. Besides, as phase distribution controls the propagation direction of the electromagnetic waves and thus plays a more important role in holographic imaging, a weak “TJU” profile could also be observed in Fig. 3g.

Thanks to the coherent detection employed at terahertz frequencies, the experimental results of phase distribution of the image can also be obtained, which are shown in Fig. 3h,i, corresponding to MAPM and MOPM, respectively. A “TJU” profile of phase distribution could be observed in Fig. 3h. However, it cannot be clearly seen in Fig. 3i as the boundary of the “TJU” could be hardly confirmed and the phase distribution is not very homogeneous, which further reflects the superior image quality of MAPM. There is little phase difference in the “TJU” image region in Fig. 3h, which matches the expectation well. In conventional phase-only computer-generated holography, many optimizing techniques including Gerchberg-Saxton method and Fresnel ping-pong algorithm are employed to increase the image quality^35^,^36^,^37^. However, the phase distribution of the image then becomes irregular, which actually means the imaged object is with rough surface leading to diffuse scattering. Compared with the traditional optimized phase-only holograms^15^,^16^,^17^,^18^,^19^,^20^,^21^, our approach could be applied in the metasurface holograms of more special and complex objects with required phase distribution, like the objects with smooth surfaces leading to regular phase distribution. Besides, as both amplitude distribution and phase distribution can be arbitrarily designed and reappeared, our approach may be used in designing holograms with large depth of field (DOF).

**Figure 5:**
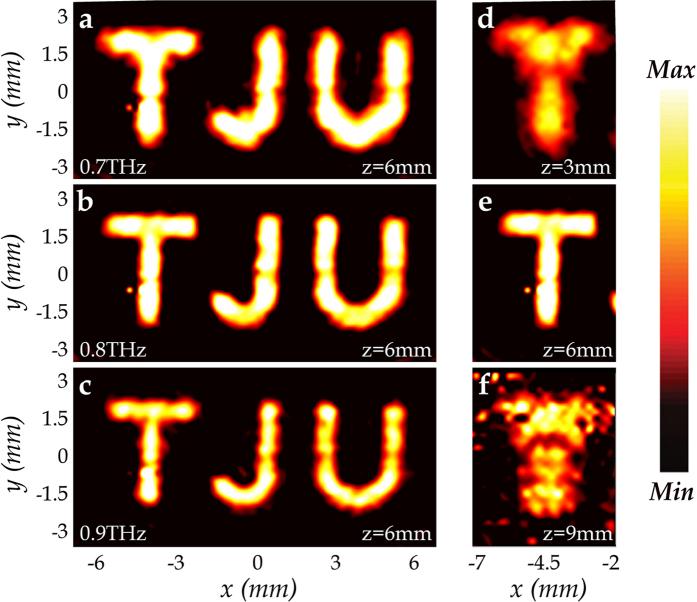
Experimental electric field amplitude distributions of MAPM at different frequencies and different locations. (**a–c**) Experimental results of the *y*-polarized electric amplitude distribution of MAPM on the image plane (*z* = 6 mm) under an *x*-polarized normal incidence at 0.7, 0.8 and 0.9 THz, respectively. (**d–f**) Experimental results of the *y*-polarized amplitude distribution of MAPM for an *x*-polarized incidence at 0.8 THz with different distances (*z* = 3, 6 and 9 mm) away from the sample.

Moreover, the experimental results of the amplitude distribution of the electric field on the image plane (*z* = 6 mm) for MAPM at different frequencies are shown in Fig. 5a–c, where a broadband and a relatively DOF performance of the metasurface could be observed. The imaging effects are limited mainly due to the fact that each frequency has its own focal plane. Along with the results in Fig. 4e,g, these results also reflect a relatively large DOF at 0.70 and 0.90 THz. Besides, the experimental amplitude distributions at different locations at 0.80 THz are also shown in Fig. 5e,f. The terahertz wave focuses on the plane of *z* = 6 mm, forming a clear sharp image of “T”, which is just as designed.

**Figure 6:**
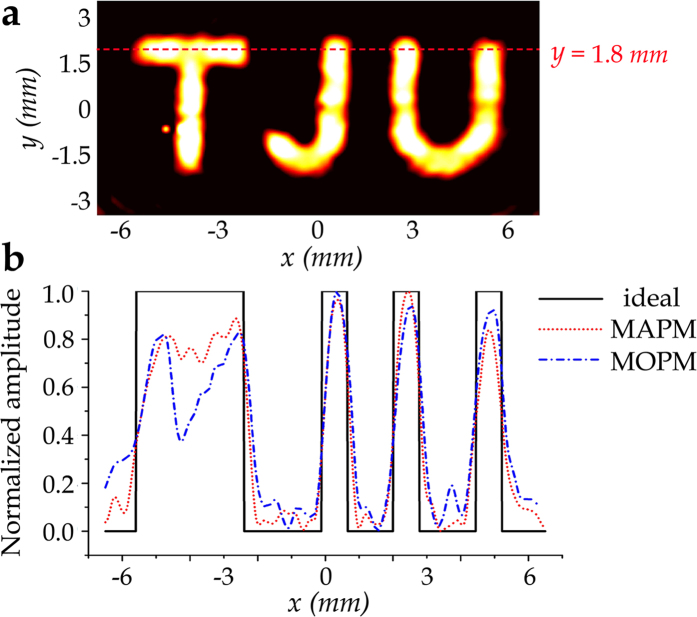
Quantitative analysis. (**a**) Illustration of the quantitative analysis setting, where the red dashed line represents the cross-section of *y* = 1.8 mm. (**b**) Normalized amplitude distributions of the cross-section of *y* = 1.8 mm, which has been marked in **a**. The black solid line shows the ideal amplitude distribution. The red dotted line and the blue dash-dotted line represent the experimental results corresponding to MAPM and MOPM, respectively.

Furthermore, we carry out the quantitative analysis to confirm the superior image quality of MAPM. We define the signal to noise ratio (SNR) as the ratio of the average intensity in the “TJU” image region to the average intensity of background noise whose sampling area is the whole rectangular region (13 × 6.5 mm^2^) apart from the “TJU” image region. The experimental SNR is 84.1 for MAPM and is 25.8 for MOPM. Besides, for a given position of *y* = 1.8 mm, normalized amplitude distributions along this cross-section are shown in Fig. 6. It could be clearly seen that the experimental result of MAPM is more homogeneous and shows lower background noise than that of MOPM, which also leads to higher resolution.

Besides the imaging quality and the broadband performance, efficiency is an important merit in holography researches. In this work, as the holography is formed by the electric field polarized orthogonal to the incidence while the explored probe can only detect one polarization, experimentally measuring the efficiency needs reassembling the probe and the sample. This will result in experimental errors which in turn influences the accuracy of the efficiency calculation. Actually, because of the excellent agreement between the experimental results and the simulation results, this efficiency calculation can be completed by simulation. The simulated efficiency by CST Microwave Studio commercial software, which is defined as the ratio between the orthogonal polarized terahertz power projected into the “TJU” region and the input power, is 19.1% for MOPM and 6.4% for MAPM, respectively. The relatively low efficiency mainly results from the transmission-type and single-layer structures. The efficiency could be easily enhanced by using the reflective type of structures or multi-layer structures as shown in some of the recent works^15^,^25^.

In conclusion, a novel broadband metasurface hologram is proposed which allows simultaneously control of the phase and the amplitude of the terahertz wave, leading to high resolution images. Compared to metasurface holograms with phase-only modulation, our amplitude-phase modulation approach results in a much better quality for the reconstructed holographic image. Besides, the design is straightforward, robust and simple in fabrication. The approach could not only be applied at terahertz frequencies, but also be referred in the visible region. The proposed ultrathin metasurface hologram paves a way to achieve high quality holograms and broadens the application space of electromagnetic wave manipulation with microstructured metasurfaces. Furthermore, this technique is promising in terahertz imaging and meticulous control over terahertz wave propagation.

## Methods

### Design of the CSRRs

The CSRRs are made from 200-nm thick aluminum with a conductivity of 3.72 × 10^7^ S·m^−1^ and patterned on a 500-μm thick silicon substrate (*ε*_si_ = 11.7)^38^. Numerical simulations on spectral response of the resonators are performed using 3D full wave simulations with the commercial software CST Microwave Studio. Periodic boundary conditions are applied in the *x* and *y* directions while perfectly-matched-layer (PML) boundary condition is applied in the *z* direction. Plane wave at Normal incidence polarized in the *x* direction is used to excite the structure. The amplitude and phase information of the scattered *y*-polarized radiation for different geometrical parameters are recorded.

### Experimental Section

The NSTM system is employed to investigate the effects of the hologram metasurfaces. Specific descriptions of the NSTM system can be found in our previous works^24^,^34^. To test the hologram characteristics of both metasurfaces on the image plane (*z* = 6 mm), the 2D electric field is detected by slow scan with a 0.2 mm step in the *x*-direction from −7 mm to + 7 mm and a 0.2 mm step in the *y*-direction from −3.5 mm to 3.5 mm. To test the hologram behaviors of MAPM at different heights, the metasurface is first moved to *z* = 3 mm and *z* = 9 mm, respectively, and the electric field is detected by the identical scan process.

## Additional Information

**How to cite this article**: Wang, Q. *et al*. Broadband metasurface holograms: toward complete phase and amplitude engineering. *Sci. Rep.* **6**, 32867; doi: 10.1038/srep32867 (2016).
